# In silico analysis of the grapefruit sRNAome, transcriptome and gene regulation in response to CTV-CDVd co-infection

**DOI:** 10.1186/s12985-017-0871-9

**Published:** 2017-10-23

**Authors:** Marike Visser, Glynnis Cook, Johan T. Burger, Hans J. Maree

**Affiliations:** 1 Department of Genetics, Stellenbosch University, Stellenbosch, South Africa; 2 Citrus Research International, Nelspruit, South Africa; 3 Agricultural Research Council, Infruitec-Nietvoorbij: Institute for Deciduous Fruit, Vines and Wine, Stellenbosch, South Africa

**Keywords:** Biotic stress, Citrus dwarfing viroid, Citrus tristeza virus, *Citrus paradisi*, High-throughput sequencing, Pathogen-response, Plant-pathogen interaction, RNA-interference, Small RNA, Transcriptome

## Abstract

**Background:**

Small RNA (sRNA) associated gene regulation has been shown to play a significant role during plant-pathogen interaction. In commercial citrus orchards co-infection of Citrus tristeza virus (CTV) and viroids occur naturally.

**Methods:**

A next-generation sequencing-based approach was used to study the sRNA and transcriptional response in grapefruit to the co-infection of CTV and Citrus dwarfing viroid.

**Results:**

The co-infection resulted in a difference in the expression of a number of sRNA species when comparing healthy and infected plants; the majority of these were derived from transcripts processed in a phased manner. Several RNA transcripts were also differentially expressed, including transcripts derived from two genes, predicted to be under the regulation of sRNAs. These genes are involved in plant hormone systems; one in the abscisic acid, and the other in the cytokinin regulatory pathway. Additional analysis of virus- and viroid-derived small-interfering RNAs (siRNAs) showed areas on the pathogen genomes associated with increased siRNA synthesis. Most interestingly, the starting position of the p23 silencing suppressor’s sub-genomic RNA generated a siRNA hotspot on the CTV genome.

**Conclusions:**

This study showed the involvement of various genes, as well as endogenous and exogenous RNA-derived sRNA species in the plant-defence response. The results highlighted the role of sRNA-directed plant hormone regulation during biotic stress, as well as a counter-response of plants to virus suppressors of RNA-silencing.

**Electronic supplementary material:**

The online version of this article (10.1186/s12985-017-0871-9) contains supplementary material, which is available to authorized users.

## Background

Plants respond to pathogen infection through a number of gene regulatory pathways. RNA-silencing is a form of regulation where double-stranded or hairpin-structured RNA precursors give rise to small RNAs (sRNAs), which become control elements for the expression of target genes [1–3]. Several types of sRNA species have been identified and characterised in plants, and their involvement in biotic stress responses have been suggested. These include microRNAs (miRNAs) [4–8], phased-siRNAs (phasiRNAs) [9–12], natural-antisense transcript siRNAs (nat-siRNAs) [12–14], repeat-associated siRNAs (rasiRNAs) [7] and tRNA-derived RNA fragments (tRFs) [15, 16].

Stem pitting is a destructive symptom in fruit crops caused by various virus species. *Citrus tristeza virus* (CTV) is a highly destructive, phloem limited, pathogen of citrus species, causing three different disease syndromes [17] of which stem pitting currently is considered the highest threat to the industry [18]. CTV belongs to the genus *Closterovirus* in the family *Closteroviridae* [19]. The ~19,300 nt genome of CTV is organised into 12 open reading frames and represents the largest known plant virus genome [20].

Due to the complex disease aetiology of CTV, which is strongly influenced by the combination of host factors and virus genotypes, the mechanism(s) behind symptom expression is poorly understood [21, 22]. A recent study, however, has suggested the involvement of different combinations of p33, p18 and p13 expression during stem pitting symptom development [18]. Three CTV RNA-silencing suppressors have been identified, namely p20, p23 and p25 (coat protein), all of which can play a role in symptom expression [23].

In addition to viruses, citrus species are also affected by viroid-infections. *Citrus dwarfing viroid* (CDVd), a member of the genus *Apscaviroid* (family *Pospiviroidae*), has been suggested for use in high-density orchards since it causes dwarfing of citrus varieties grafted onto *Poncirus trifoliata* (*P. trifoliata*) and its hybrids, without reducing production [24–26]. The fact that citrus species are often co-infected with CTV and viroids prompted a recent study which investigated the co-infection of CTV and CDVd in their respective indicator plants, Mexican lime and etrog citron [27]. A host-specific increase in the accumulation of CDVd was observed in the presence of CTV, along with the synthesis of CDVd-associated sRNAs. These observations were ascribed mainly to the involvement of the CTV-encoded silencing suppressor, p23. It is also interesting to note that the co-infection did not affect symptom expression under their experimental conditions [27].

Understanding the mechanisms involved in pathogen infection and symptom expression provides the information required to study and potentially engineer disease resistance. In this study, we used a next-generation sequencing (NGS) approach to investigate the plant responses to the co-infection of CTV and CDVd in two commercial grapefruit (*Citrus paradisi*) cultivars, on both the sRNA and transcriptome level. Our results highlighted the association of CTV-CDVd co-infection with the expression of various genes and sRNA species, these include sRNAs involved in the regulation of plant hormones.

## Methods

### Sample preparation

Grapefruit plants (cultivars ‘Marsh’ and ‘Star Ruby’) on ‘Carrizo’ citrange rootstocks were bark-inoculated from an asymptomatic *Citrus sinensis* (sweet orange) plant that was confirmed to be infected with CTV (genotype T3) and CDVd by RT-PCR using previously described protocols [28, 29]. Briefly, bark-inoculation was performed by patch-grafting two bark chips of the source plant to each grapefruit scion, once the scion was approximately 7 mm thick. All plants were inoculated at the same height. After inoculation, the scions were cut back approximately 10 cm above the inoculation point. One shoot of the new growth was allowed to grow from the top bud. Un-inoculated plants served as healthy controls.

Total RNA was extracted from the phloem material of three replicates of healthy and infected plants of each cultivar following an adapted CTAB method from Carra et al. [30]. Virus and viroid status was confirmed using the above-mentioned RT-PCR assays.

### Next-generation sequencing and data preparation

Total RNA extracted from each sample was sent for sequencing on an Illumina HiSeq instrument (Fasteris, Geneva, Switzerland). Two libraries per sample were prepared and sequenced. An sRNA library was generated from 18 to 30 nt size-selected RNA and sequenced in a 1 × 50 nt run, as well as a transcriptome library generated from ribo-depleted total RNA and sequenced in a 2 × 125 nt run. Adapter sequences were trimmed from the data using cutadapt [31]. Fastx-toolkit [32] was used to remove all low quality reads from the sRNA data, while Trimmomatic [33] was used to filter and trim the transcriptome data for quality. sRNA reads, 18–26 nts in length and transcriptome reads, 20 nts and longer were retained for further analyses.

Virus and viroid infection status of samples were confirmed bioinformatically with the mapping of virus-derived siRNA (vsiRNA) and viroid-derived siRNA (vd-siRNA) derived NGS data against the respective genomes as described below. BLASTn [34] analysis of assembled contigs (described below) against NCBI’s nt database was used to exclude the possible presence of any other viruses or viroids from the data. The viral status of samples were further verified using CTV-specific e-probes [35, 36].

### Grapefruit transcriptome-assembly, differential expression analysis and natural-antisense transcript (NAT) identification

Trinity [37, 38] was used to assemble the transcriptome data into contigs, applying default parameters. Transcript differential expression analysis was performed using the DESeq2 [39] method in Trinity. Gene ontology analyses were performed using Trinity and Blast2GO [40].

Assembled contigs were used to identify NATs by aligning contigs to each other, using BLASTn [34] to identify overlapping regions of 50 nts and longer with 100% identity. Duplex formation of the overlaps was validated with UNAfold [41].

### Grapefruit sRNA identification, differential expression analysis and target prediction

To identify novel miRNAs and phased (PHAS) transcripts within grapefruit, all sRNA datasets were simultaneously submitted to ShortStack [42, 43], with default parameters. The assembled contigs served as template for precursor identification. DESeq2 was used to analyse the differential expression of all unique sRNA sequences. Differentially expressed sRNAs were characterised based on their comparison to different sRNA species as described below.

The predicted novel miRNAs, along with the plant entries in miRBase 21 [44–47], as well as the predicted phasiRNAs (sRNAs that fell into a dominant phasing register), served as database for the identification of differentially expressed sRNAs, which were miRNAs or phasiRNAs, respectively. Differentially expressed sRNAs were also mapped, using Bowtie [48], onto the identified PHAS transcripts, the overlapping regions of the NATs, plant repetitive sequences in RepBase [49, 50], and the sequences of plant tRNAs in the PlantRNA database [51], to identify PHAS-associated sRNAs (not in phase with the dominant phasing register), nat-siRNAs, rasiRNAs and tRFs, respectively.

psRNATarget [52] was used, applying default settings, to predict targets for the differentially expressed sRNAs using the assembled transcriptome as a list of potential targets.

### Pathogen-derived sRNA analysis

vsiRNAs (associated with CTV) and vd-siRNAs (associated with CDVd) were identified by mapping the sRNA reads, using Bowtie, onto the CTV-T3 (Accession No. KC525952) and CDVd (Accession No. AF184149) genomes allowing a single, or no mismatches, respectively.

## Results

### Symptom expression in grapefruit

**Fig. 1:**
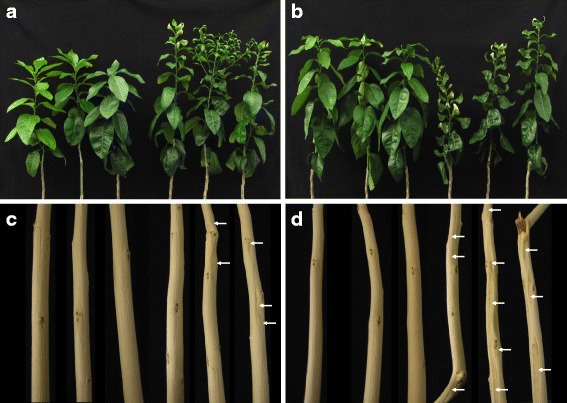
CTV-CDVd co-infected plants. Three healthy followed by three CTV-CDVd co-infected (**a**) ‘Marsh’ and (**b**) ‘Star Ruby’ plants. A representative stem sample (after bark removal) is given below each plant (**c** and **d**)

Healthy ‘Marsh’ and ‘Star Ruby’ grapefruit plants were co-infected with CTV and CDVd, using an asymptomatic (CTV and CDVd infection confirmed) sweet orange plant as source. The co-infection was confirmed with RT-PCRs and supported through NGS read mapping analysis (shown below). No additional viruses or viriods were identified in the NGS data. ‘Star Ruby’ plants showed more distinct leaf cupping and stem pitting symptoms than ‘Marsh’ plants (Fig. 1).

### NGS data preparation

sRNA and transcriptome NGS datasets were generated for each RNA sample extracted from phloem tissue. The raw data ranged from 14,740,885 to 22,862,616 sRNA reads and 12,726,094 to 16,410,600 transcriptome read-pairs per sample, while the high-quality datasets ranged from 8,550,133 to 12,715,167 sRNA reads (after quality filtering) and 12,012,340 to 15,458,590 transcriptome read-pairs (after quality filtering and trimming) per sample.

### Grapefruit transcriptome assembly and differential expression

High-quality reads from all the transcriptome datasets were combined and de novo assembled into transcripts (contigs). Altogether 214,371 transcripts were generated, which could be grouped into 120,991, Trinity-defined, “genes”.

**Fig. 2:**
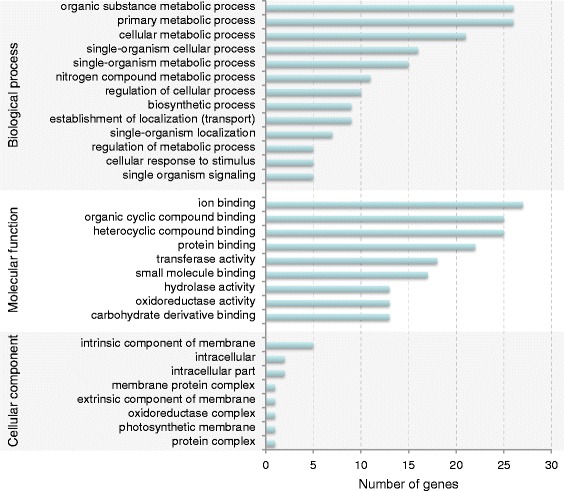
Gene ontology classification of differentially expressed genes. Barr-graph illustrating the number of differentially expressed genes assigned to biological process, molecular function and cellular component gene ontology terms (level 3)

Differential expression analysis was subsequently performed to identify transcripts involved in CTV-CDVd co-infection. In ‘Marsh’ and ‘Star Ruby’ 675 and 1204 transcripts, respectively, showed altered expression (Additional file 1: Tables S1 and S2). The results also identified 154 “genes” for which at least one transcript were differentially expressed between healthy and infected plants, across both grapefruit varieties (Additional file 1: Table S3). According to similarity searches, these included 21 potential disease response genes, as well as 60 membrane and 10 photosystem-associated genes, highlighted through gene ontology (GO) analysis (Fig. 2, Additional file 1: Tables S4-S6 and Additional file 2: Figures S1-S3). Many transcripts were however homologous to hypothetical or uncharacterised citrus proteins, resulting in 33% of the differentially expressed “genes” remaining unidentified.

### Endogenous sRNA identification and regulation

Combined analysis of the transcript and sRNA data predicted miRNAs from 60 grapefruit miRNA genes (*MIRs*) that were expressed in at least one of the samples (Additional file 3: Table S7). For 38 of these *MIRs,* neither of the mature miRNAs predicted, were represented by any mature plant-derived miRNA in miRBase. In addition to the predicted miRNAs, reads with sequences identical to 216 known plant miRNAs were also present in the data (Additional file 3: Table S8). Significant phasing was also seen in 7268 transcripts (called PHAS transcripts), producing 63,943 phasiRNAs in total, which were in phase with the dominant phasing register. To facilitate the identification of nat-siRNAs, transcripts were subjected to NAT identification. The duplex formation of 25,378 transcripts, predicted to be part of one or more NAT pair, were validated in silico. The overlapping regions were extracted for subsequent nat-siRNA analysis.

**Fig. 3:**
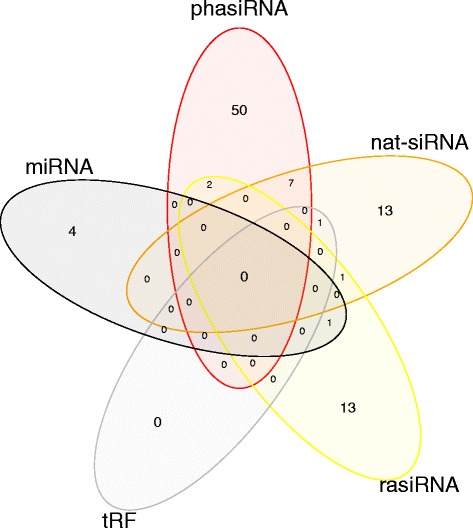
Species identification of differentially expressed sRNAs. Venn diagram illustrating the overlapping sRNA species (miRNA, phasiRNA, nat-siRNA, rasiRNA, tRF) identities of differentially expressed sRNAs

Differential expression analysis revealed 761 sRNAs with altered expression levels resulting from pathogen infection (Additional file 4: Tables S9 and S10). Of these, 577 were variety-specific, while 184 showed differential expression across varieties (Additional file 4: Table S11). These sRNAs were characterised based on sequence homology to either the predicted, or other plant miRNAs, predicted phasiRNAs, PHAS transcripts, the overlaps of NATs, as well as plant repetitive DNA-regions and tRNAs. While a number of sRNAs could be classified as nat-siRNAs (22), miRNAs (five), rasiRNAs (17) or tRFs, the majority (59) of differentially expressed sRNAs were phasiRNAs. Some could potentially be classified into more than one sRNAs species, for example two sRNAs derived from a repetitive region and seven sRNAs derived from the overlapping region of a NAT that were all processed in a phased manner (Fig. 3).

To determine the biological role that sRNAs play during CTV-CDVd co-infection, all differentially expressed sRNAs were subjected to in silico target prediction. Only three sRNAs (one uncharacterised and two derived from PHAS transcripts) showed significant inverse-regulation with respect to their predicted target transcripts (Additional file 5: Table S12), one of which was across the two varieties, and the other two specific to ‘Star Ruby’. Homology searches identified the across-variety sRNA target as a chloroplastic Magnesium-chelatase subunit ChlH (CHLH) and the ‘Star Ruby’-specific sRNA targets as Cytokinin dehydrogenase 6 (CKX) and a hypothetical protein.

### Pathogen-derived sRNAs

**Fig. 4:**
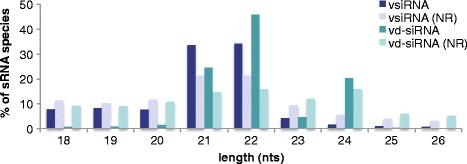
Size-distribution of vsiRNA and vd-siRNA reads. Histogram illustrating the number of vsiRNA and vd-siRNA reads, 18 nt to 26 nt in length, from the infected samples, all (redundant) as opposed to unique (non-redundant, NR), as a percentage of the vsiRNA and vd-siRNA reads in this size-range respectively

**Fig. 5:**
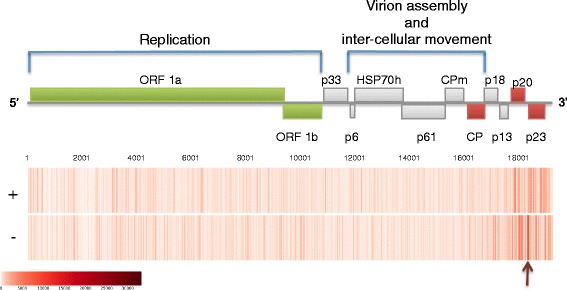
Distribution of vsiRNA reads along the CTV genome. vsiRNA profile generated from sRNA reads depicted as heat maps showing the reads that mapped onto the positive (+) or negative (−) strand of CTV. A schematic representation of the genome above the heat maps illustrates the genomic position of the vsiRNA reads. The start of the p23 subgenomic RNA, which forms an vsiRNA hotspot, is indicated with an arrow

Virus-derived siRNAs (vsiRNAs) were found associated with 97% of the CTV-T3 reference genome. The majority of the vsiRNAs were 21 or 22 nts in length (Fig. 4). The distribution of the sRNA reads on the genome showed an increase in vsiRNAs mapping towards the 3′ end of the virus (Fig. 5). A prominent hotspot for sRNA synthesis was observed on the negative-strand at the sub-genomic RNA initiation site of p23.

**Fig. 6:**
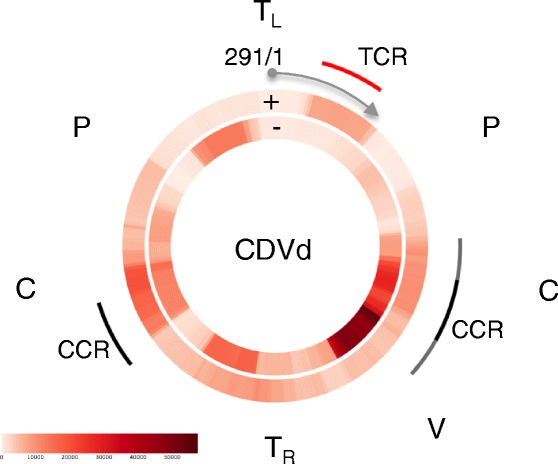
Distribution of vd-siRNA reads along the CDVd genome. vd-siRNA profile generated from sRNA reads depicted as heat maps showing the reads that mapped onto the positive (+) or negative (−) strand of CDVd. A vd-siRNA hotspot was formed on the negative-strand of the viroid, overlapping the central and variable regions. C, central domain; P, pathogenic domain; TCR, terminal conserved region; CCR, central conserved region; T_L_, terminal left domain; T_R_, terminal right domain; V, variable domain

In the case of CDVd, viroid-derived siRNAs (vd-siRNAs) were mostly 22 nts in length, followed by reads 21 and 24 nts in length (Fig. 4), and covered the complete viroid genome (Fig. 6). A specific area on the negative-strand of the viroid, overlapping the central and variable regions, gave rise to an abundance of sRNAs, indicating a potential target area.

## Discussion

In the field, citrus plants are often subjected not only to CTV infection but also to the co-infection with viroids. It is therefore necessary to understand this combined pathogen interaction with the plant host in order to improve disease resistance strategy design. In this study, healthy ‘Marsh’ and ‘Star Ruby’ grapefruit plants developed leaf cupping and stem pitting symptoms after being co-infected with CTV (genotype T3) and CDVd. These symptoms were not present in the co-infected sweet orange plants, which served as inoculation source. Although the T3 genotype is associated with increased stem pitting, previous studies have shown that CTV isolate is not the only determinant of symptom expression, but that host species also plays a role [18, 21, 22]. The mechanism(s) that drive the severity and host-specific symptom expression remains to be elucidated.

An sRNA and transcriptome next-generation sequencing approach was followed to study the gene-regulatory pathways involved in the CTV-CDVd co-infection of grapefruit. To compensate for the limited genomic information available for grapefruit, the transcriptome data were de novo assembled to generate a case-specific grapefruit transcriptome. Since the assembled transcripts represented both coding and non-coding transcripts, they were used to identify both the precursors and targets of sRNAs.

As potential precursor source, the transcripts were first used for miRNA discovery. The miRNA registry, miRBase, currently holds no entries for grapefruit. Here we report on the identification of 60 grapefruit *MIR* genes along with their mature miRNAs, based on in silico prediction analysis. Many of the other sRNA reads represented homologous plant miRNAs. Once more grapefruit genome information becomes available, these homologous sequences may still prove to be true miRNAs, expressed from grapefruit *MIR* genes. In addition to miRNAs, phasiRNAs and PHAS transcripts were also identified, based on the assembled transcriptome, along with NATs that form the precursors of nat-siRNAs.

Many diverse “genes” were found differentially regulated in response CTV-CDVd co-infection across both grapefruit varieties. Similar to the results of previous studies on the infection of either CTV [53–57] or CDVd [58] in different citrus species and in *P. trifoliata*, grapefruit metabolic, disease response, structural and phytohormone-related pathways seem to be affected by the co-infection. Genes with known involvement in plant disease responses were mostly grouped into leucine-rich repeat (which include tobacco mosaic virus resistance protein N-like) and ankyrin repeat-containing protein coding genes. The number of chloroplast-associated genes with altered expression supports the hypothesis that the CTV-CDVd co-infection influences the photosynthetic pathways of grapefruit, which was previously shown for CTV infection in sweet orange [56, 57] and Mexican lime [54, 59].

Many members of different endogenous sRNA species, such as miRNAs, phasiRNAs, nat-siRNAs, rasiRNAs and tRFs also showed variation in expression resulting from the co-infection. Target prediction was performed to determine the biological role of the differentially expressed sRNAs. Despite many transcripts and sRNAs showing differential expression resulting from co-infection, an inverse-regulation was only seen for three sRNA-transcript pairs. The apparent disconnect between sRNAs and their predicted targets could be due to a number of factors. First, the target prediction software used, was designed specifically for miRNA and phasiRNA target prediction and may therefore not be as effective for other sRNA species. Target prediction models remain to be developed for the other species, following the characterisation of their mechanism(s) of action. Second, although sRNA action may lead to the indirect inhibition of protein expression, the presence of any (cleaved or uncleaved) target transcript-related reads in the transcriptome dataset will count towards transcript-associated reads during differential expression analysis of the genes. Last, the relatively low read counts associated with many of the predicted target transcripts could have influenced the statistical significance of the differential expression analysis.

The combined results from this study suggested the sRNA-directed gene regulation of plant hormone pathways during CTV-CDVd co-infection in grapefruit. The expression of chloroplastic Magnesium-chelatase subunit ChlH (CHLH) showed inverse-regulation with respect to that of its regulating sRNA, across both ‘Marsh’ and ‘Star Ruby’ plants. In Arabidopsis, CHLH plays a role in chlorophyll biosynthesis, the expression of photosynthesis-related proteins, as well as abscisic acid (ABA) signal regulation [60]. CHLH was shown to repress the expression of the disease response *WRKY40* gene in the ABA signalling pathway [61]. Therefore, unsurprisingly, *WRKY40* showed inverse-regulation to that of CHLH resulting from the co-infection. The involvement of the ABA pathway in plant-virus response has previously been described [62–64], and includes the restriction, to some extent, of virus movement through callose deposition [62]. In addition, ABA was shown to contribute to virus-resistance through the regulation of Argonauts [64]. The sRNA-directed regulation of CHLH during the CTV-CDVd co-infection could therefore potentially have a down-stream effect on virus resistance through ABA regulation. Cytokinin dehydrogenase 6 (CKX), on the other hand, is involved in the irreversible down-regulation of cytokinins [65–67], and showed inverse-regulation with respect to that of its regulating sRNA in ‘Star Ruby’ plants. Cytokinins can stimulate plant defence response upon pathogen infection [68], and may lead to either an enhancement in resistance [69–71], or susceptibility to viral infections [72]. The observed down-regulation of CKX may lead to an increase in cytokinin levels, contributing to the grapefruit defence response. A tissue-specific up- or down-regulation of CKX resulting from virus infection was also recently observed in Arabidopsis roots and shoots, respectively [73].

Pathogen-derived sRNA profiles have been found to vary upon different infection. The majority of the vsiRNAs, derived from CTV, were 21 or 22 nts in length, which is common for vsiRNAs produced by many plants, including citrus [8, 74]. The vsiRNAs also seem to favour the 3′ end of the virus. While this increase towards the 3′ end of CTV was observed before, not all genotype-host combinations showed the same pattern [8, 74]. Factors that could influence patterns of vsiRNA synthesis include; a host-specific response, the virus genome secondary structure, virus gene expression and host-specific suppression of virus proteins.

Interestingly, when looking at the vsiRNA profile of CTV, a highly prominent hotspot was observed at the sub-genomic RNA initiation site of p23, associated with the negative strand, which indicates an area targeted by the host-response. p23 is known to play a major role in CTV-host interaction, especially as a suppressor to counter the host’s RNA-silencing response [23]. The targeting of p23 by the host therefore adds another layer to the CTV-host interaction.

Recent studies have also investigated the plant siRNA response to viroid infection [75–79]. While the majority of CDVd-derived vd-siRNAs were 22, 21 or 24 nts in length respectively, the observed size-distribution may be tissue dependent [76, 77]. As was seen for CTV, an sRNA hotspot was observed associated with an area on the negative-strand of CDVd. A similar hotspot was previously observed for another member of the same genus, *Potato spindle tuber viroid*, in tomato [75, 78]. The implication of the sRNA targeting of this specific area of the viroid remains to be elucidated.

## Conclusions

In silico analysis of the transcriptome and sRNAs generated in response to CTV and CDVd co-infection in grapefruit, suggested the involvement of sRNAs in the regulation of plant hormone pathways in infected plants. Further analysis revealed regions within both the CTV and CDVd genomes that form hotspots for vsiRNA and vd-siRNA synthesis. The specific vsiRNA hotspot associated with p23, suggests a first report of potential vsiRNA counter-response by the plant to the p23-silencing suppressor of CTV. An exciting future prospect for these pathogen-derived sRNAs could be their application in disease resistance strategies.

## Additional files

Additional file 1: Table S1. Results for the transcript differential expression analysis of ‘Marsh’ plants. Transcripts were considered to be differentially regulated as a result of infection if a |log2 fold change| > =1 and padj < =0.05 were observed. **Table S2.** Results for the transcript differential expression analysis of ‘Star Ruby’ plants. Transcripts were considered to be differentially regulated as a result of infection if a |log2 fold change| > =1 and padj < =0.05 were observed. **Table S3.** Results for the gene differential expression analysis across both ‘Marsh’ and ‘Star Ruby’ plants. **Table S4.** Biological process based characterisation of genes differentially expressed across both grapefruit varieties. **Table S5.** Molecular function based characterisation of genes differentially expressed across both grapefruit varieties. **Table S6.** Cellular component based characterisation of genes differentially expressed across both grapefruit varieties. (XLSX 392 kb)

Additional file 2: Figure S1. Biological process based network of genes differentially expressed across both grapefruit varieties. **Figure S2.** Molecular function based network of genes differentially expressed across both grapefruit varieties. **Figure S3.** Cellular component based network of genes differentially expressed across both grapefruit varieties. (PDF 2159 kb)

Additional file 3: Table S7. miRNA prediction results. **Table S8.** Homologous plant miRNA results. (XLSX 26 kb)

Additional file 4: Table S9. Results for the sRNA differential expression analysis of ‘Marsh’ plants. sRNAs were considered to be differentially regulated as a result of infection if a |log2 fold change| > =1 and padj < =0.05 were observed. **Table S10.** Results for the sRNA differential expression analysis of ‘Star Ruby’ plants. sRNAs were considered to be differentially regulated as a result of infection if a |log2 fold change| > =1 and padj < =0.05 were observed. **Table S11.** Results for the sRNA differentially expressed across both ‘Marsh’ and ‘Star Ruby’ plants. sRNAs were considered to be differentially regulated as a result of infection if a |log2 fold change| > =1 and padj < =0.05 were observed. (XLSX 106 kb)

Additional file 5: Table S12. Differential expression analysis results showing sRNAs with anti-correlated expression to their targets. (XLSX 53 kb)

## Abbreviations

ABA: abscisic acid
CDVd: Citrus dwarfing viroid
CHLH: chloroplastic magnesium-chelatase subunit ChlH
CKX: cytokinin dehydrogenase 6
CTV: Citrus tristeza virus
MIR: miRNA gene
miRNA: microRNA
NAT: natural-antisense transcript
nat-siRNA: natural-antisense transcript siRNA
nts: nucleotides
*P. trifoliata*: *Poncirus trifoliata*
PHAS: phased transcript
phasiRNA: phased-siRNA
rasiRNA: repeat-associated siRNA
siRNA: small-interfering RNA
sRNA: small RNA
tRF: tRNA-derived RNA fragment
vd-siRNA: viroid-derived siRNA
vsiRNA: virus-derived siRNA
