# Supplementary figures and images for: In silico analysis of the grapefruit sRNAome, transcriptome and gene regulation in response to CTV-CDVd co-infection
Source: Virol J. 2017 Oct 23;14:200. doi: 10.1186/s12985-017-0871-9 (PMC5651572; doi:10.1186/s12985-017-0871-9)

Figure S1

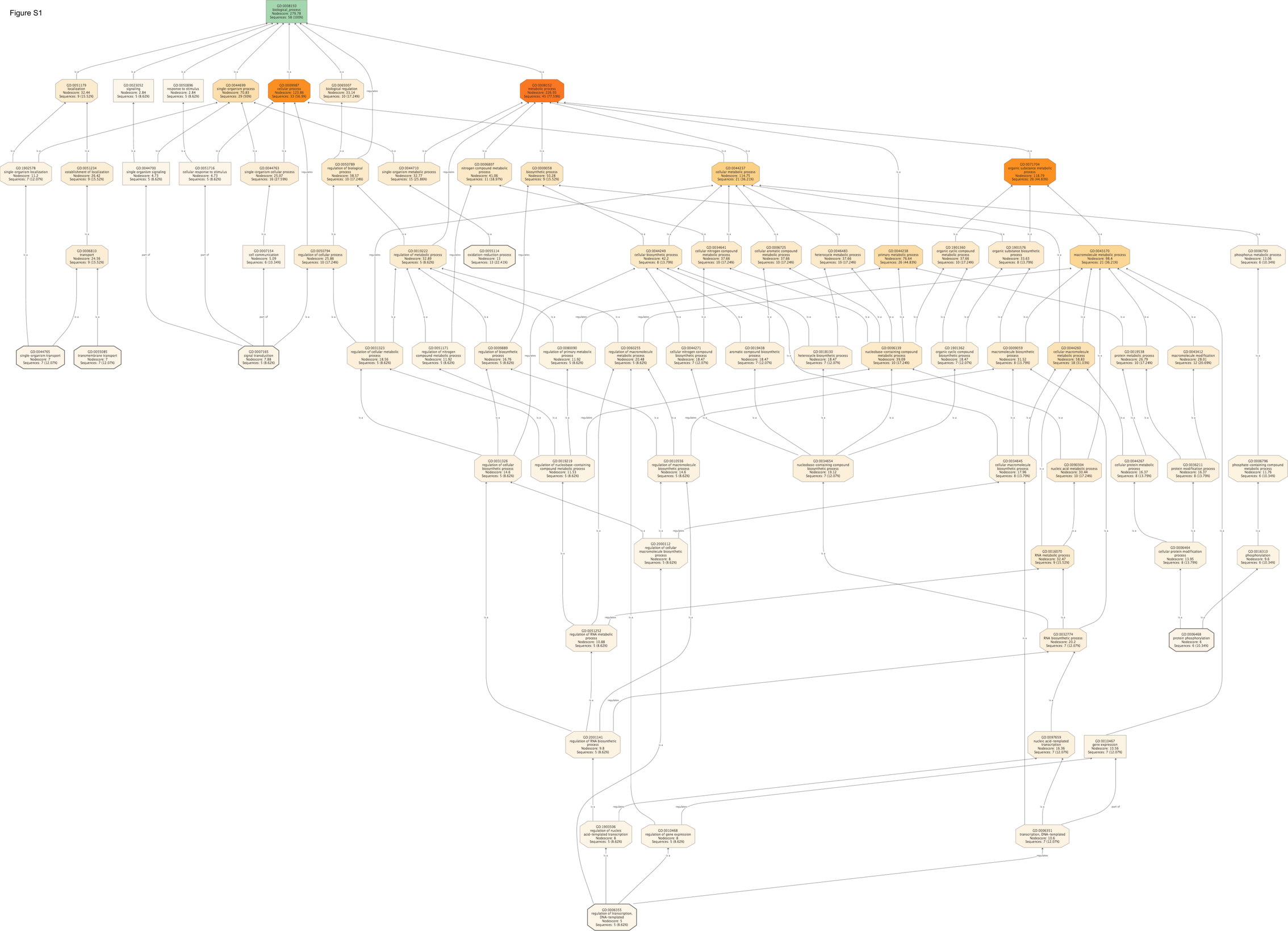

Figure S2

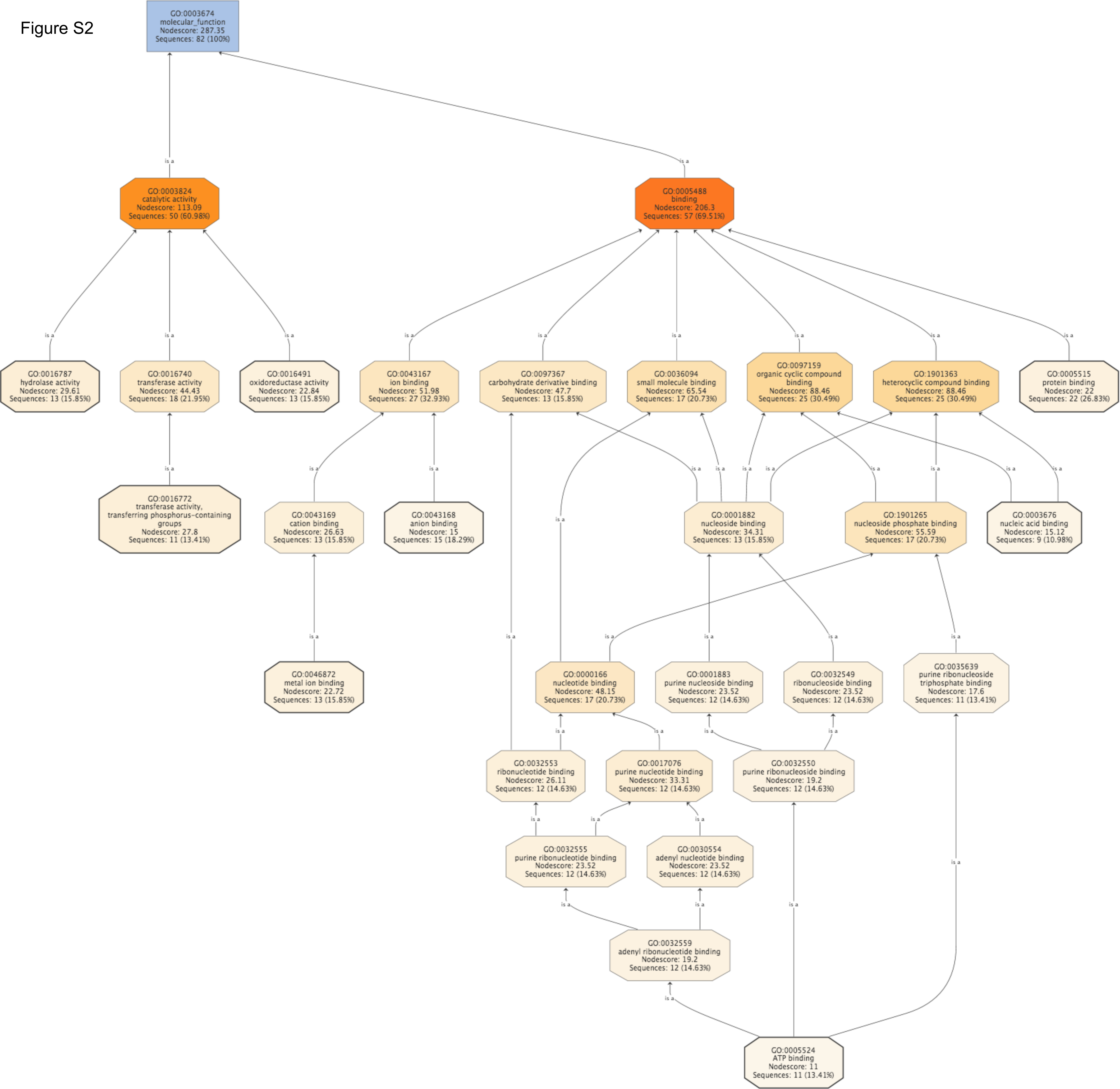

Figure S3

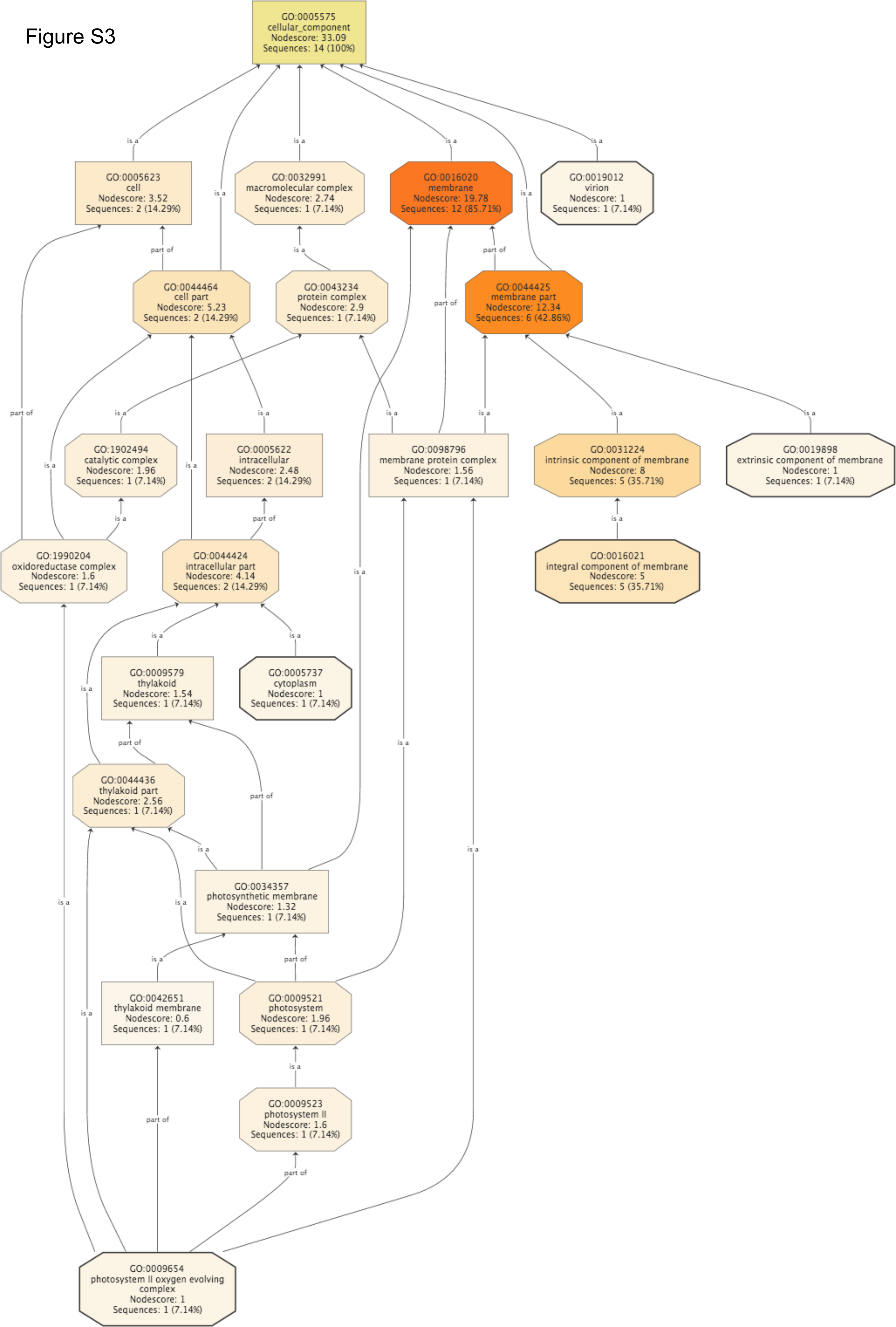

Supplement: Supplementary file 2 — Biological process based network of genes differentially expressed across both grapefruit varieties. Figure S2. Molecular function based network of genes differentially expressed across both grapefruit varieties. Figure S3. Cellular component based network of genes differentially expressed across both grapefruit varieties. (PDF 2159 kb) [file 12985_2017_871_MOESM2_ESM.pdf]
